# The Role of *Dactylis Glomerata* and Diesel Oil in the Formation of Microbiome and Soil Enzyme Activity

**DOI:** 10.3390/s20123362

**Published:** 2020-06-13

**Authors:** Agata Borowik, Jadwiga Wyszkowska, Mirosław Kucharski, Jan Kucharski

**Affiliations:** Department of Microbiology, University of Warmia and Mazury in Olsztyn, 10-727 Olsztyn, Poland; agata.borowik@uwm.edu.pl (A.B.); miroslaw.kucharski@sloik.net (M.K.); jan.kucharski@uwm.edu.pl (J.K.)

**Keywords:** PHAs, bacterial taxa, soil enzymes, *Dactylis glomerata*, phytoremediation

## Abstract

The global demand for petroleum contributes to a significant increase in soil pollution with petroleum-based products that pose a severe risk not only to humans but also to plants and the soil microbiome. The increasing pollution of the natural environment urges the search for effective remediation methods. Considering the above, the objective of this study was to determine the usability of *Dactylis glomerata* for the degradation of hydrocarbons contained in diesel oil (DO), as well as the effects of both the plant tested and DO on the biochemical functionality and changes in the soil microbiome. The experiment was conducted in a greenhouse with non-polluted soil as well as soil polluted with DO and phytoremediated with *Dactylis glomerata*. Soil pollution with DO increased the numbers of microorganisms and soil enzymes and decreased the value of the ecophysiological diversity index of microorganisms. Besides, it contributed to changes in the bacterial structure at all taxonomic levels. DO was found to increase the abundance of *Proteobacteria* and to decrease that of *Actinobacteria*, *Acidobacteria*, *Chloroflexi*, *Gemmatimonadetes* and *Firmicutes*. In the non-polluted soil, the core microbiome was represented by *Kaistobacter* and *Rhodoplanes*, whereas in the DO-polluted soil, it was represented by *Parvibaculum* and *Rhodococcus*. In soil sown with *Dactylis glomerata*, gasoline fraction (C_6_–C_12_) degradation was higher by 17%; mineral oil (C_12_–C_35_), by 9%; benzene, by 31%; anthracene, by 12%; chrysene, by 38%; benzo(a)anthracene, by 19%; benzo(a)pyrene, by 17%; benzo(b)fluoranthene, by 15%; and benzo(k)fluoranthene, by 18% than in non-sowed soil. To conclude, *Dactylis glomerata* proved useful in degrading DO hydrocarbons and, therefore, may be recommended for the phytoremediation of soils polluted with petroleum-based products. It has been shown that the microbiological, biochemical and chemical tests are fast and sensitive in the diagnosis of soil contamination with petroleum products, and a combination of all these tests gives a reliable assessment of the state of soils.

## 1. Introduction

What all organisms need to exist is a continuous flow of energy [1,2]. Some of them derive energy from sun rays while others do so from organic and inorganic compounds [3,4,5]. This diversity makes them able to convert various pollutants [6], including those contained in petroleum-based products [7], which consequently affects the succession of microorganisms in the natural environment [6,8]. Hydrocarbons [7,9,10,11], heavy metals [12,13,14,15] and pesticides [16,17] are a severe threat to the natural environment due to their high accumulation potential and high toxicity in the soil ecosystem [18]. Hydrocarbons can be more easily removed than heavy metals, because they serve as a source of carbon and energy to microorganisms [19,20] and thus can be thoroughly degraded [21,22,23]. According to Yang et al. [24], Patowary et al. [25], and Bidja et al. [10], the microorganisms able to transform hydrocarbons and, by this means, to remove them from soil include, among others, *Nocardia soli*, *Rhodococcus erythropolis*, *Bacillus pumilus* and *Bacillus cereus* or *Serratia marcescens* and *Raoultella ornithinolytica*. According to Obi et al. [26] and Xu et al. [20], bacteria of the following genus may be useful in soil detoxification for PAH: *Achromobacter*, *Arthrobacter*, *Enterobacter*, *Klebsiella*, *Kocuria*, *Mesorhizobium* and *Pseudomonas*. Essential in the removal process for petroleum-derived hydrocarbons is not only their degradation but also modification of their hydrophobic and hydrophilic properties by biosurfactant-producing microorganisms, e.g., *Bacillus methylotrophicus*, *Bacillus licheniformis*, *Proteus vulgaris*, *Proteus mirabilis*, *Serratia marcescens*, *Sphingomonas paucimobilis*, and *Micrococcus kristinae* [27,28].

Despite the ability of certain microorganisms to use hydrocarbons as energetic substrates [20,26], these chemicals pose a severe threat to other soil organisms and plants [29], and to the entire trophic chain [30]. Environmental exposure to the effects of various groups of xenobiotics can trigger changes in the functioning of a microbial population [31]. This, in turn, aggravates the threat posed to the optimal functioning of soil ecosystems, especially considering the recently increased environmental pollution [32]. All this has prompted a search for inexpensive methods that would aid the degradation of toxic compounds in soils [33]. One of the most effective methods enabling the counteracting of the effects of soil pollution is bioremediation [7,8,22,33,34], and one of its techniques is phytoremediation [29,35,36,37]. Cheap and effective technologies for the remediation of contaminated soils are currently in high demand. Biodegradation and phytostabilization are environmentally friendly, and therefore, the search for plants resistant to pollution is justified. The absorption of pollutants by plants, the translocation and accumulation of pollutants, their stabilization in the root zone and the transformation of organic pollutants by rhizosphere microorganisms are extremely valuable [38]. Phytoremediation is considered one of the most environmentally friendly methods. In situ biological methods used for soil reclamation rely on the synergistic interaction of plants and associated microorganisms [29,35,36,37]. Microorganisms supported by organic plant substrates can contribute to the significant degradation of compounds contained in petroleum substances [19]. Therefore, the search for plants and microorganisms effective in improving the quality of the soil environment poses an essential challenge to science and environmental engineering. They ought to be investigated, considering their function, as they cannot be plants grown for food but rather are useful for energetic purposes [19,37,39,40], e.g., *Dactylis glomerata* var. Bepro, which was used in our study for the phytoremediation of soil polluted with diesel oil. The goals of this study were to determine the usability of *Dactylis glomerata* for the degradation of diesel oil hydrocarbons and to investigate the effects of both the plant tested and diesel oil on the biochemical functionality and changes in soil microbiome at various taxonomic levels. The knowledge gained from this study may prove useful in the biotechnology for removing petroleum-based products from the soil, because the potential of plant rhizosphere microorganisms, aided by nutritional substrates produced by plant roots, can significantly contribute to the remediation of soils and polycyclic aromatic hydrocarbon (PAH) degradation.

## 2. Materials and Methods

### 2.1. Study Object

**Soil**. The study was conducted with Eutric Cambisol soil having the following composition: 74.93% of sand fraction (grain diameter: 0.05–2 mm), 22.85% of silt fraction (grain diameter: 0.002–0.05 mm), and 2.22% of clay fraction (grain diameter: < 0.002 mm). The soil had high contents of phosphorus (93.68 mg of available P kg^−1^ d.m. soil) and potassium (141.10 mg of available K kg^−1^ d.m. soil), and a medium content of magnesium (42 mg of available Mg kg^−1^ d.m. soil). It contained 0.62 g N kg^−1^ d.m. soil and 9.30 g C_org_ kg^−1^ d.m. soil. Its pH in 1 mol KCl dm^−3^ was 6.7, and its exchangeable capacity was 60.40 mmol (+) kg^−1^ d.m. soil. The soil was collected from a depth of 0 to 20 cm of the arable lands from the Olsztyn Lake District situated in the north-east of Poland, within the province of the Eastern Baltic-Belarusian Lowland, which is part of the Eastern European Plain (NE Poland, 53.7161N, 20.4167E).

**Diesel oil**. The study was conducted with BP diesel oil (DO) with Active technology, composed of C_10_–C_28_ hydrocarbons (over 90%) [41]. The choice of this DO was driven by its increasing use in Poland and other EU Member States [42]. 

**Plant**. The test plant used for phytoremediation was orchard grass (*Dactylis glomerata*) of Bepro variety. It is an energy grass characterized by a biomass production of 11 to 13 Mg d.m. ha^−1^. It withstands drought and low temperatures [43], is relatively resistant to diseases [40] and exhibits high capability for adapting to varying weather conditions. *Dactylis glomerata* is a plant highly resistant to PAH contamination. It shows growth on virtually any type of soil. Its dense bundle root system allows soil penetration over a large area [19,44,45].

### 2.2. Study Design

Because the study assumed the detailed identification of the microbiome of soil polluted with a petroleum-based product, it was performed under strictly controlled conditions in a greenhouse in Kick–Brauckman polyethylene pots. This prevented pollutant migration to the natural environment. The experiment was performed with 4 replications and established in 4 series: (1) non-polluted and non-sown soil (C), (2) non-polluted soil sown with *Dactylis glomerata* (Dg), (3) non-sown soil polluted with diesel oil (DO), and (4) soil polluted with diesel oil and sown with *Dactylis glomerata* (DgDO). 

The soil was sieved through a screen with a mesh diameter of 1 cm, thoroughly mixed, and transferred to pots. Afterward, its 9 kg portions were prepared (for each pot), supplemented with the following macroelements, per 1 kg of soil—80 mg N in the form of CO(NH_2_)_2_, 20 mg P in the form of KH_2_PO_4_, 40 mg K in the form of KCl and KH_2_PO_4_, and 10 mg Mg in the form of MgSO_4_ · 7H_2_O—and thoroughly mixed. The soil intended for the first and second series of the experiment was placed in pots, whereas the soil intended for the third and fourth series was first polluted with diesel oil at an amount of 7 cm^3^ kg^−1^ soil, mixed, and then placed in the pots. In all experimental series, the soil was moisturized to 60% of the capillary water capacity. After 7 days since the experiment had been established, 24 seeds of *Dactylis glomerata* were sown into the soil from Series 2 and 4. The grass was cut 3 times: on Day 45, Day 75 and Day 105 of the experiment. Soil moisture was monitored throughout the study period, and water losses were compensated with distilled water. Microbiological, biochemical and chemical analyses were carried out immediately after the 3rd grass cutting, i.e., on the 105th day of the experiment.

### 2.3. Methodology of Microbiological Analyses

A serial dilution method was used to determine counts of organotrophic bacteria (Org), actinobacteria (Act), and fungi (Fun) in all the soil samples according to the procedure presented in work by Borowik et al. [46]. Simultaneously, DNA was extracted from soil samples using the “Genomic Mini AX Soil+” kit, and its presence was confirmed with Real-Time PCR in an Mx3000P thermocycler (Stratagene), using an SYBR Green dye (A&A Biotechnology, Gdynia, Poland) as a fluorochrome. The PCR mixture contained 1055F (5’-ATGGCTGTCGTCAGCT-3’) and 1392R (5’-ACGGGCGGTGTGTAC-3’) primers, while the specific primers 341F/785R were used to amplify the selected region and prepare the metagenomic data library. The next-generation sequencing was carried out using an Ilumina MiSeq platform (Illumina Inc., San Diego, California, USA), with the paired-end (PE) technology, 2 *×* 250 bp, using a v2 Illumina kit. The bioinformatic analysis of the hypervariable region V3–V4 of the 16S rRNA gene, after filtering out the chimeric and incomplete sequences, was conducted using the QIIME packages based on reference sequence databases GreenGenes v13_8, by Genomed S.A. Warsaw, Poland.

### 2.4. Physical, Chemical and Biological Properties of Soil 

The activity of dehydrogenases was determined according to the procedure provided by Öhlinger [47]; the activities of urease, acid phosphatase, alkaline phosphatase, urease, β-glucosidase, and arylsulfatase, according to Alef and Nannipieri [48]; and the activity of catalase, according to Johnson and Temple [49]. The substrates used in enzymatic activity analyses, activity units, and conditions of analyses were described in our previous work by Borowik et al. [46]. Soil samples were analysed for contents of benzines (C_6_–C_12_), mineral oil (C_12_–C_35_), aromatic volatile hydrocarbons (BETX), and PAHs—2 rings (naphthalene), 3 rings (anthracene), 4 rings (chrysene, benzo(a)antracene), 5 rings (benzo(a)pyrene, benzo(b)fluoranthene, benzo(k)fluoranthene), and 6 rings (benzo(ghi)perylene, indeno(123-cd)pyrene. The PAH content was determined by Weeseling company (Cracow, Poland) on an Agilent 7890A–5975C gas chromatograph with a mass spectrometer, equipped with an EI/CI ion source, according to the following ISO standards: ISO 18,287 [50], EN ISO 16,703 [51] and EN ISO 22,155 [52]. The procedures used to determine contents of organic carbon, total nitrogen, available phosphorus, available potassium, and available magnesium and to analyze soil pH, exchangeable capacity and soil fraction size distribution were presented in the work by Borowik et al. [46]. The organic carbon content was determined with the Tiurin method according to Nelson and Sommers [53]; total nitrogen content, with the Kjeldahl method according to ISO 11,261 [54]; content of P_available_ and K_available_, with the method of Egner-Riehm [55]; Mg_available_ content, with atomic absorption spectrometry according to Schlichting et al. [56]; granulometric composition, with the aerometric method PN-R-04032 [57] and ISO 11,464 [58]; pH_KCl_, with the potentiometric ISO 10,390 [59] method; and exchangeable capacity, with the Kappen method according to Klute [60].

### 2.5. Statistical Computations and Analysis

The determined counts of bacteria and fungi were used to compute the colony development index (CD) and the ecophysiological diversity index (EP), according to De Leij et al. [61]. Relative abundance was visualized using the STAMP 2.1.3. software, based on the two-sided test of statistical hypotheses-G-test (w/Yates’) + Fisher’s, with the interval confidence method-Asymptotic with CC [62]; the Circos 0.68 package used to present genomic data in the circular system; and the RStudio v1.2.5033 software [63], v3.6.2 system [64], and gplots library [65] were used to generate the heat map. The relative abundance of the bacteria of each taxon was presented from the data for which the contribution was higher than 1%. The indices of diesel oil effect (I_DO_) and of plant effect (I_DR_) on the activity of soil enzymes as well as the resistance index (RS) of *Dactylis glomerata* to diesel oil were computed as well [66]. The contents of benzines (C_6_–C_12_), mineral oil (C_12_–C_35_), aromatic volatile hydrocarbons (BETX) and PAHs determined in the soil directly after its pollution with DO and 105 days after its pollution allowed the computation of the degree of their degradation. Both the counts of soil microorganisms; the activities of soil enzymes; and the values of the CD, EP, I_DO_ and I_DR_ indices were used to calculate homogenous groups with the Tukey test at a significance level of P = 0.05, using the Statistica 13.1 package [67]. The results of the analysis of variance (ANOVA) are shown in Table S1.

## 3. Results

### 3.1. Counts and Diversity of Microorganisms in the Soil

**Figure 1:**
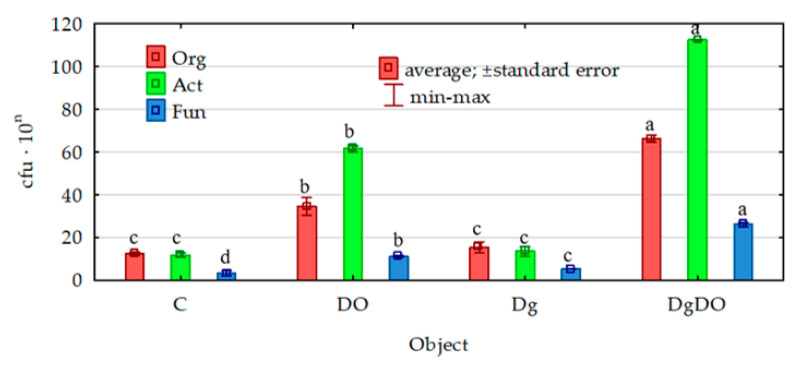
Effect of diesel oil (DO) and *Dactylis glomerata* (Dg) on the numbers of organotrophic bacteria and actinobacteria, 10^9^ cfu kg^−1^ d.m. soil, and fungi, 10^7^ cfu kg^−1^ d.m. soil. Homogeneous groups denoted with letters (a–d) were calculated separately for each of the microorganisms. C—non-polluted and non-sown soil, Dg—non-polluted soil sown with *Dactylis glomerata*, DO—non-sown soil polluted with diesel oil, DgDO—soil polluted with diesel oil and sown with *Dactylis glomerata*.

Diesel oil significantly stimulated the proliferation of all the microorganisms studied (Figure 1). In the non-sown soil, the number of organotrophic bacteria increased by 180%; that of actinobacteria, by 429%; and that of fungi, by 225%. The cultivation of orchard grass offered convenient conditions, promoting the development of all the analyzed groups of microorganisms. This was confirmed in both the non-polluted soil (increase in population numbers from 16% in the case of actinobacteria to 47% in the case of fungi) and the polluted soil (increase in populations numbers of 83% for actinobacteria and 136% for fungi). 

**Figure 2:**
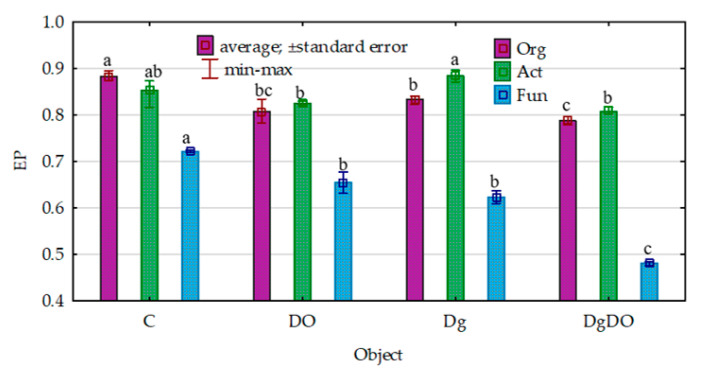
Effect of diesel oil (DO) and *Dactylis glomerata* (Dg) on the ecophysiological diversity index (EP) of the microorganisms. Homogeneous groups denoted with letters (a–c) were calculated separately for each of the microorganisms. C—non-polluted and non-sown soil, Dg—non-polluted soil sown with *Dactylis glomerata*, DO—non-sown soil polluted with diesel oil, DgDO—soil polluted with diesel oil and sown with *Dactylis glomerata.*

**Figure 3:**
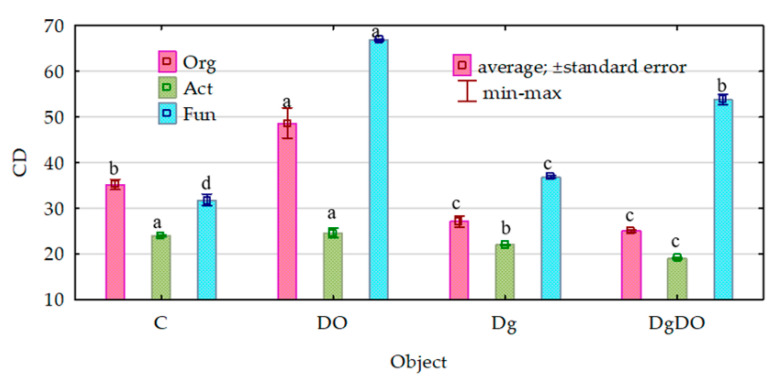
Effect of diesel oil (DO) and *Dactylis glomerata* (Dg) on the colony development index (CD). Homogeneous groups denoted with letters (a–d) were calculated separately for each of the microorganisms. C—non-polluted and non-sown soil, Dg—non-polluted soil sown with *Dactylis glomerata*, DO—non-sown soil polluted with diesel oil, DgDO—soil polluted with diesel oil and sown with *Dactylis glomerata.*

Despite the strong promotion of the microorganisms caused by soil pollution with diesel oil, the values of their ecophysiological diversity index (EP) decreased significantly (Figure 2). Theoretically, the EP index can range from 0 to 1, and the colony development index (CD), from 10 to 100. The greater the value of the EP index, the greater the diversity of microorganisms. For organotrophic bacteria, they decreased from 0.88 to 0.81 in the non-sown soil and from 0.83 to 0.79 in the sown soil; for actinobacteria, from 0.85 to 0.83 and from 0.88 to 0.81, respectively; and for fungi, from 0.72 to 0.65 and from 0.62 to 0.48, respectively. Nevertheless, the values of the colony development index (CD) were significantly lower in the non-polluted soil and the DO-polluted soil sown with *Dactylis glomerata* than in the non-sown soil (Figure 3). This means that slow-growing bacteria (strategy-r) dominated in sown soil, whereas fast-growing bacteria (strategy-K) dominated in non-sown soil.

**Figure 4:**
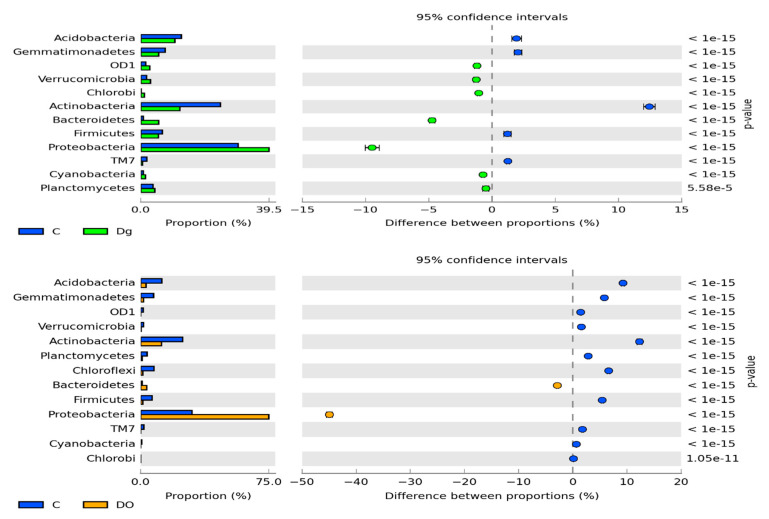

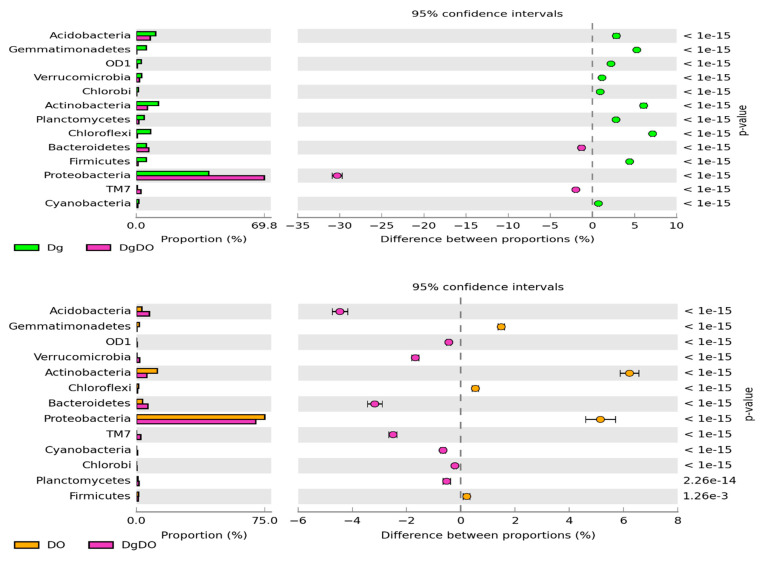
The relative abundance of the dominant bacterial type in the soil with a difference between proportions ≥ 1%. C—non-polluted and non-sown soil, Dg—non-polluted soil sown with *Dactylis glomerata*, DO—non-sown soil polluted with diesel oil, DgDO—soil polluted with diesel oil and sown with *Dactylis glomerata.*

The prevailing taxon at the phylum level was represented by *Proteobacteria* (Figure 4). In the non-polluted and non-sown soil, they accounted for 28.8% of total bacteria, and in the soil sown with *Dactylis glomerate*, for 36.8% of total bacteria. Soil pollution with DO significantly increased their abundance to 74.3% in the non-remediated soil and to 67.2% in the soil remediated with *Dactylis glomerata*. Such a high abundance of *Proteobacteria* caused lower operational taxonomic unit (OTU) numbers of most of the other phyla. It was especially noticeable for *Actinobacteria*, *Acidobacteria*, *Chloroflexi*, *Gemmatimonadetes* and *Firmicutes*, whose OTU numbers in the DO-polluted non-sown soil were lower by 11.4%, 8.7%, 6.3%, 5.5% and 5.2%, respectively, compared to in the non-polluted and non-sown soil. Slightly lesser changes were observed in the soil remediated with *Dactylis glomerata*. 

**Figure 5:**
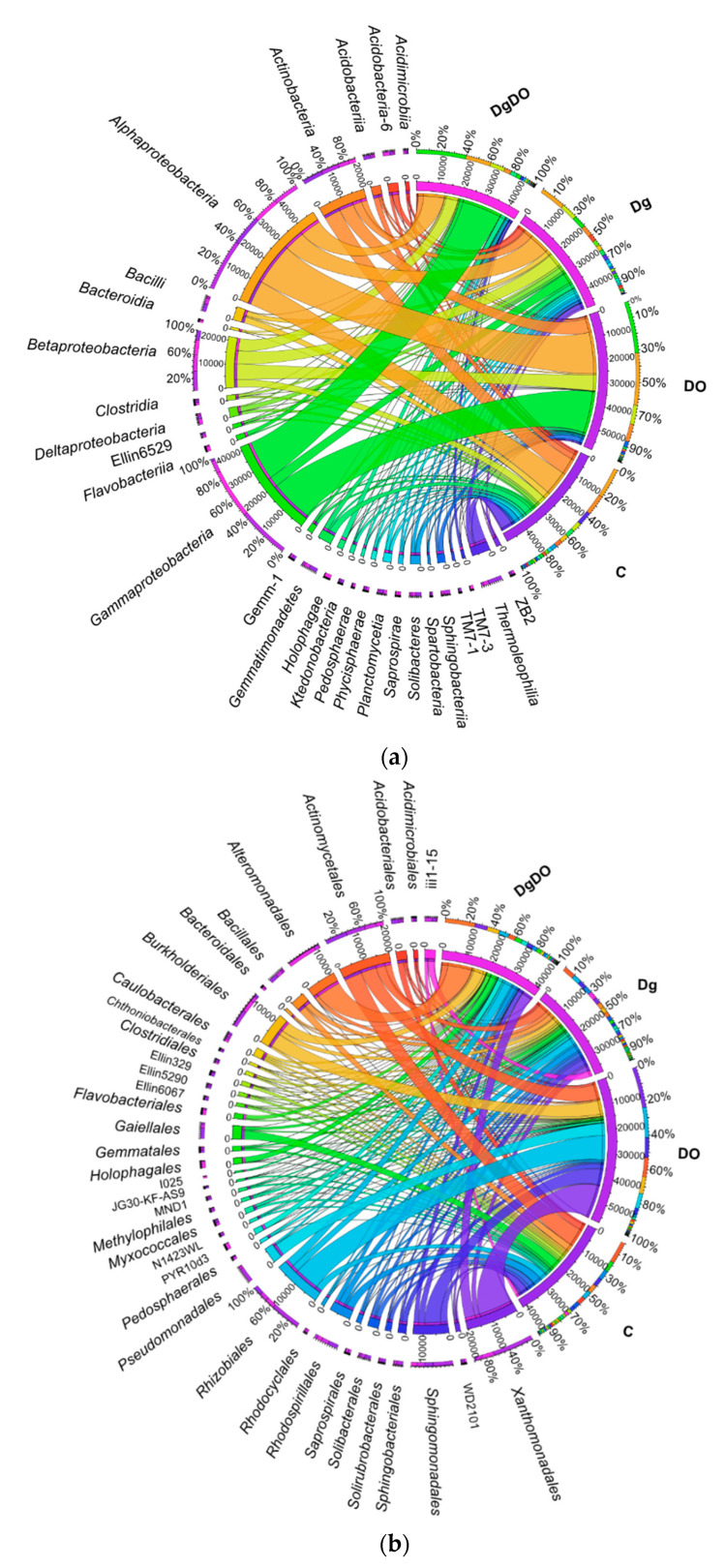
The relative abundance of the dominance class (**a**) and order (**b**) of bacteria in soil with a difference between proportions ≥1%. C—non-polluted and non-sown soil, Dg—non-polluted soil sown with *Dactylis glomerata*, DO—non-sown soil polluted with diesel oil, DgDO—soil polluted with diesel oil and sown with *Dactylis glomerata*.

Changes observed already at the phylum level in the bacterial structure were also reflected in the other taxa (Figure 5a,b). Regardless of the soil cultivation variant, among the phylum *Proteobacteria,* bacteria of the class *Alphaproteobacteria* predominated in the non-polluted soil (17.42%), and *Gammaproteobacteria*, in the DO-polluted soil (33.76%). Orchard grass cultivation contributed to higher OTU numbers of three (*Betaproteobacteria, Deltaproteobacteria, Gammaproteobacteria*) out of the four identified classes. In turn, diesel oil increased the abundance of *Gammaproteobacteria* by as much as 25 times in the non-sown soil, and 3.9 times in the soil sown with *Dactylis glomerata*. This spectacular increase in the OTU number of the *Gammaproteobacteria* class in the non-sown soil was caused mainly by the bacteria from orders *Pseudomonadales* and *Xanthomonadales*, and in the sown soil, by the *Alteromonadales, PYR10d3* and *Xanthomonadales* bacteria. Interesting dependencies were also observed for less abundant taxa. The cultivation of *Dactylis glomerata* in the DO-polluted soil increased the population numbers of bacteria from the following classes—*Acidobacteria-6* (o_*iii1-15*), *Holophagae* (o_*Holophagales*), *Acidimicrobiia* (o_*Acidimicrobiales*), *Saprospirae* (o_*Saprospirales*), *Clostridia* (o_*Clostridiales*), *TM7-3* (o_*I025*) and *Pedosphaerae* (o_*Pedosphaerales*)—and decreased the numbers of *Acidobacteriia* (o_*Acidobacteriales*), *Solibacteres* (o_*Solibacterales*), *Actinobacteria* (o_*Actinomycetales*), *Thermoleophilia* (o_*Solirubrobacterales*), *Sphingobacteriia* (o_*Sphingobacteriales*), *Ktedonobacteria* (o_JG30-KF-AS9), *Bacilli* (o_*Bacillales*), *Gemmatimonadetes* (o_*Ellin5290*) and *Spartobacteria* (o_*Chthoniobacterales*). Despite the diversified responses of the mentioned taxa to soil pollution with diesel oil and to the cultivation of *Dactylis glomerata*, we have managed to identify the so-called core bacteriobiome, typical of the soils from all the variants studied. It was constituted by bacteria belonging to *Actinomycetales (c_Actinobacteria)*; *Burkholderiales (c_Betaproteobacteria)*; *Caulobacterales*, *Rhizobiales*, *Rhodospirillales*, and *Sphingomonadales (c_Alphaproteobacteria)*; and *Xanthomonadales (c_Gammaproteobacteria)*. 

**Figure 6:**
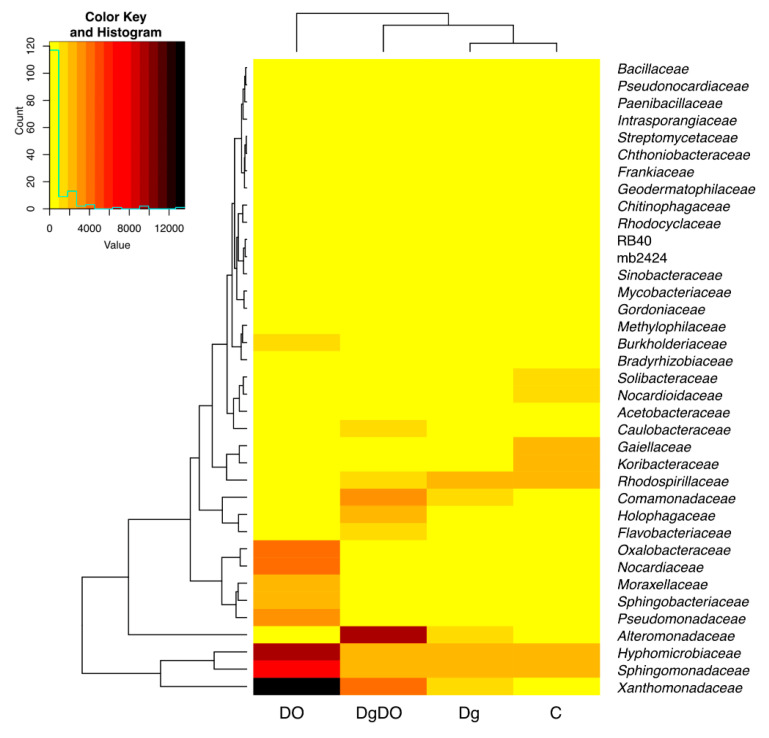
Heat map and relationships of the operational taxonomic unit (OTU) numbers of 37 bacterial families in the soil with a difference between proportions ≥1%. C—non-polluted and non-sown soil, Dg—non-polluted soil sown with *Dactylis glomerata*, DO—non-sown soil polluted with diesel oil, DgDO—soil polluted with diesel oil and sown with *Dactylis glomerata*.

**Figure 7:**
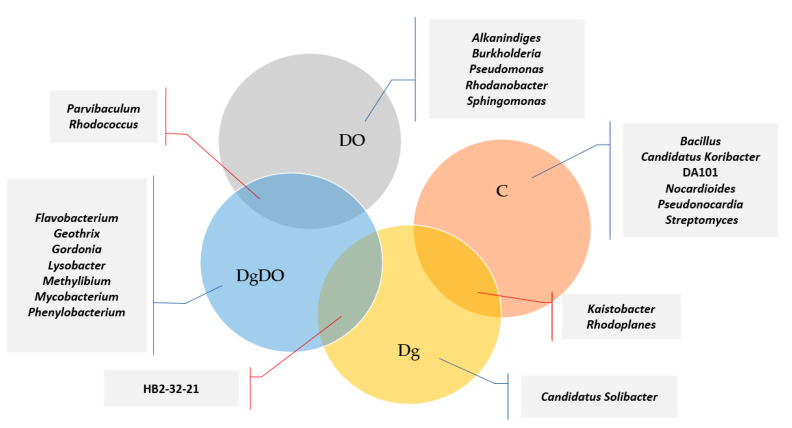
Venn diagram showing unique and common genera of bacteria, based on OTU > 1%.

Diesel oil and orchard grass also modified bacterial diversity at the family level (Figure 6). In the non-sown soil, the highest OTU numbers were noted for the bacteria from the *Gaiellaceae* (2644), *Sphingomonadaceae* (2577) and *Rhodospirillaceae* (2298) families. After soil pollution with DO, the prevailing families included *Xanthomonadaceae* (13,560 OTU), *Hyphomicrobiaceae* (9343 OTU), *Sphingomonadaceae* (6589 OTU), *Oxalobacteraceae* (4159 OTU) and *Nocardiaceae* (4184 OTU), and after sowing *Dactylis glomerata* into the soil not polluted with DO, the prevailing family turned out to be *Sphingomonadaceae* (2243 OTU). In turn, orchard grass cultivation on the DO-polluted soil promoted the development of bacteria from the *Alteromonadaceae* (9931 OTU), *Xanthomonadaceae* (4101 OTU) and *Comamonadaceae* (3538 OTU) families. Therefore, the high OTU numbers noted for the families identified in the DO-polluted non-sown soil were mainly affected by the *Rhodanobacter, Parvibaculum, Sphingomonas* and *Rhodococcus* genera. Considering the OTU numbers higher than 1% ascribed to individual bacterial genera, worthy of notice is that more genera were classified in the DO-polluted than in the non-polluted soil (Figure 7). The core bacteria were identified for each soil variant. In total, 24 various genera were identified of which only two, *Parvibaculum* and *Rhodococcus*, were characteristic for the DO-polluted soils, and another two, *Kaistobacter* and *Rhodoplanes*, for the non-polluted soils, and finally, HB2-32-21 was characteristic for the soils sown with *Dactylis glomerata*. The bacteria representing *Bacillus*, *Candidatus Koribacter*, *DA101*, *Nocardioides*, *Pseudonocardia* and *Streptomyces* occurred as the bio-core only in the non-polluted non-sown soil; *Candidatus Solibacter*, in the non-polluted soil sown with *Dactylis glomerata*; *Alkanindiges*, *Burkholderia*, *Pseudomonas*, *Rhodanobacter* and *Sphingomonas*, in the non-sown but DO-polluted soil; and finally, *Flavobacterium*, *Geothrix*, *Gordonia*, *Lysobacter*, *Methylibium*, *Mycobacterium*, and *Phenylobacterium*, in the DO-polluted soil sown with *Dactylis glomerata*. 

### 3.2. Activity of Soil Enzymes

Diesel oil enhanced the activities of all soil enzymes, whereas orchard grass only enhanced the activity of dehydrogenases, catalase and arylsulfatase (Table 1). Dehydrogenase, catalase, β-glucosidase and arylsulfatase activity were positively correlated with the number of microorganisms (Table S2). A positive correlation was also found between dehydrogenase, urease, β-glucosidase, alkaline phosphatase and the Shannon index at the genus level.

**Figure 8:**
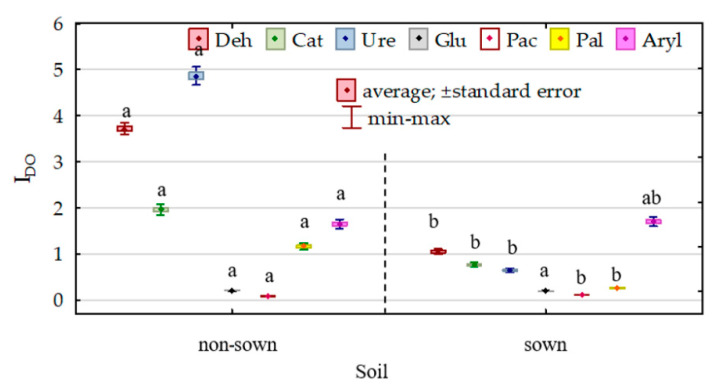
Index of the effect of diesel (I_DO_) on soil enzyme activity. Homogeneous groups denoted with letters (a,b) were calculated separately for each enzyme.

**Figure 9:**
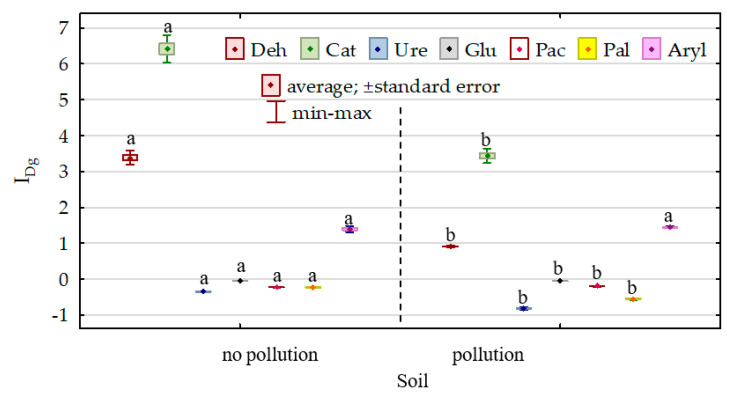
Index of the effect of *Dactylis glomerata* (I_Dg_) on soil enzyme activity. Homogeneous groups denoted with letters (a,b) were calculated separately for each enzyme.

The modification of the biochemical properties of soil due to its pollution with DO and its remediation with *Dactylis glomerata* was reflected in the indices of the effect of diesel oil (IF_DO_) and indices of the effect of orchard grass (IF_Dg_) on the activities of individual enzymes (Figure 8 and Figure 9). The effect of diesel oil on the activities of dehydrogenases, catalase, urease, β-glucosidase and alkaline phosphatase was significantly stronger in the non-remediated soil, whereas its effect on the activities of acidic phosphatase and arylsulfatase was stronger in the soil remediated with *Dactylis glomerata*. Considering the IF_DO_ values noted for the non-remediated soil, the enzymes are ordered as follows (starting from the highest value of the index)—Ure (4.863) > Deh (3.710) > Cat (1.959) > Aryl (1.647) > Pal (1.163) > Glu (0.206) > Pac (0.081)—whereas in the soil remediated with *Dactylis glomerata*, they are ordered as follows: Aryl (1.701) > Deh (1.050) > Cat (0.769) > Ure (0.647) > Pal (0.262) > Glu (0.192) > Pac (0.112). The values of the index (IF_Dg_) of the effect of soil remediation with *Dactylis glomerata* were highly varied. Both in the non-polluted and DO-polluted soils, the IF_Dg_ values determined for dehydrogenases, catalase and arylsulfatase were positive, whereas these determined for urease, β-glucosidase, acidic phosphatase and alkaline phosphatase were negative.

### 3.3. Degradation of Hydrocarbons

Chemical compounds that are constituents of diesel oil were degraded in the soil at various rates, depending on the soil cultivation variant (Table 2). In the non-sown soil, hydrocarbons degraded more slowly than in the soil sown with *Dactylis glomerata*. After sowing the soil with *Dactylis glomerata* grass, the degradation of the compounds contained in diesel oil was 13% higher than in non-sown soils. On average, over 90% degradation was noted for xylenes, ethylbenzene, toluene and naphthalene; 87–57% degradation was noted for anthracene, benzines (C_6_–C_12_), benzene and mineral oils (C_12_–C_35_); 36–26% degradation was noted for benzo(a)pyrene, chrysene, benzo(k)fluoranthene, benzo(a)anthracene and benzo(b)fluoranthene; and 11% degradation was noted for indeno(123-cd)pyrene.

### 3.4. Response of Dactylis Glomerata to Diesel Oil

**Figure 10:**
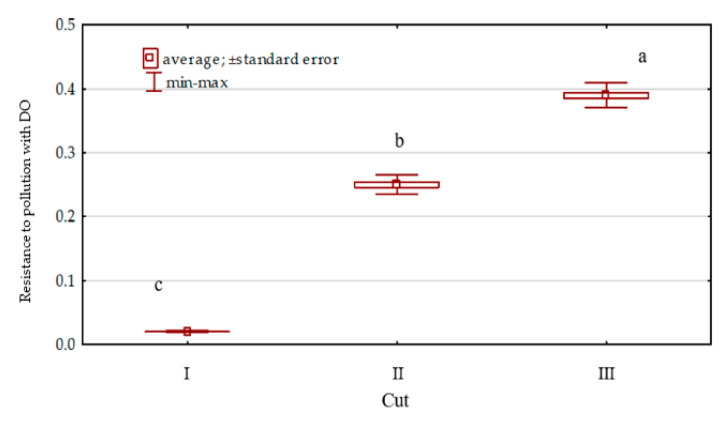
*Dactylis glomerata* resistance to soil pollution with diesel oil (DO) soil contamination.

Diesel oil added to the soil significantly impaired the growth and development of *Dactylis glomerata* (Figure 10). It had an especially adverse effect at the initial stage of orchard grass growth, as shown by a very low value of the index of its resistance (RS = 0.02) to soil pollution. With time, *Dactylis glomerata* was observed to better adapt to the adverse conditions. The index of its resistance (RS) to soil pollution with DO increased successively from 0.02 (cut I) to 0.39 (regrowth III).

## 4. Discussion

### 4.1. Counts and Diversity of Microorganisms in the Soil

Soil pollution with diesel oil as well as the sowing of non-polluted and polluted soils with *Dactylis glomerata* had a significant impact on the diversity and composition of the soil microbiome. The results of our experiment are consistent with findings reported by Yang et al. [2], Estendorfer et al. [39], Gu et al. [68], Kumar et al. [69], Gałązka et al. [70], Delgado-Baquerizo et al. [71] and Wemheuer et al. [72]. Microorganisms colonizing the soil environment are responsible for most of the geochemical processes, including nutrient metabolism and energy transfer, whereas the adhesion of microbial cells in the soil, their growth and their development are determined by the granulometric composition of soil; availability of nutrients; and contents of carbon, water, pollutants and substances secreted by plants [6,7,29]. The statistical analysis of the data obtained in our study demonstrated that soil pollution with diesel oil significantly stimulated the abundance of all microorganisms tested, whereas the cultivation of *Dactylis glomerata* enhanced their proliferation. This strong effect of diesel oil on the promotion of microorganisms was reflected in significantly decreased values of the ecophysiological diversity index (EP) of both bacteria, actinobacteria, and fungi.

The metagenomic data obtained demonstrated *Proteobacteria* taxa to be the most abundant in all the soil samples. Hence, our results did not diverge from most of the literature data [2,39,68,70,71]. Soil pollution with diesel oil reduced the abundance of *Actinobacteria*, *Acidobacteria*, *Chloroflexi*, *Gemmatimonadetes* and *Firmicutes*. Next to *Proteobacteria*, *Acidobacteria*, *Actnobacteria*, and *Planctomycetes*, Delgado-Baquerizo et al. [71] successively identified *Verrucomicrobia Bacteroidetes*, *Gemmatimonadetes*, *Firmicutes*, *Armatimonadetes*, TM7 and WS2. They claimed the repeatability of bacterial phylotypes in the soils of the world to be low. Changes in the diversity of microorganisms in the soil polluted with petroleum-based products were also confirmed by Kumar et al. [69]. In the present study, the samples of DO-polluted soil were also dominated by *Proteobacteria*, followed by *Bacteroidetes* and *Actinobacteria*. In turn, apart from *Proteobacteria*, Yang et al. [2] also demonstrated the presence of *Actinobacteria* and *Firmicutes* as well as *Bacteroidetes* and *Chloroflexi*. In our study, *Chloroflexi* and *Gemmatimonadetes* were also identified in the soil apart from the bacteria mentioned above. This was probably due to the trophic changes in the soil exposed to the pressure of diesel oil. For this reason, *Chloroflexi*—being facultative anaerobes—could faster adapt to the adverse conditions [68,73]. Bacteria present in the soils polluted with petroleum-based products aid the survivability of aerobic heterotrophs [73].

A surprising finding was *Gemmatimonadetes*’ response to soil contamination with diesel oil. According to DeBruyn [74], they represent approximately 2% of the soil bacterial population. In the non-polluted soil analyzed in our study, they accounted for 3–5%, whereas after soil pollution with DO, their contribution in the soil microbiome decreased to barely 0.16–1.4%. These results are in opposition to the findings reported by Gałązka et al. [70], who proved the positive response of *Gemmatimonadales* to petroleum-based products.

Changes in the bacterial structure in soils polluted with diesel oil are determined not only by seasonal variations in the availability of nutrients and C and N resources but also by interactions between the root system of plants and bacteria [39], the moisture and oxygen contents in the soil [75], and the species of the grown plant [72]. Modifications of the structure of microbial communities, observed in our study, were also demonstrated in the experiment conducted by Estendorfer et al. [39], where the soil sown with *Dactylis glomerata* was dominated by *Sinobacteriaceae*, *Hyphomicrobiaceae*, *Comamonadaceae*, *Chitinophagaceae* and *Xanthomonadaceae*, whereas the non-sown soil was dominated by *Chitinophagaceae*, *Hyphomicrobiaceae* and *Sinobacteriaceae*.

In the present study, among the prevailing phyla worthy of notice are the genera of *Alkanindiges*, *Rhodococcus*, *Burkholderia*, *Candidatus Koribacter*, *Candidatus Solibacter*, *Geothrix*, *Lysobacter*, *Methylibium*, *Mycobacterium*, *Parvibaculum*, *Pseudomonas*, *Phenylobacterium*, *Rhodanobacter*, *Rhodococcus*, *Sphingomonas*, HB2-32-21, *Kaistobacter* and *Burkholderia* but also bacteria from the *Bacillus* and *Flavobacterium* genera. According to Yang et al. [2], Raju et al. [76], Mukherjee et al. [77] and Røberg et al. [78], they are able to degrade petroleum-based products. However, as reported by Islam et al. [73] and Hemkemeyer et al. [79], the bacterial diversity in the soil environment is determined by the abundance of energy and nutrient resources, whereas the availability of these resources may be diminished for certain bacteria by the adverse effects of diesel oil on the physical properties of soil.

### 4.2. Activity of Soil Enzymes

The basic source of soil enzymes is microorganisms and, to a lesser extent, plants and other organisms. The enzyme activity is positively correlated with the abundance and structure of microorganisms [12,14,21,80]. Therefore, apart from microorganisms, soil enzymes are sensitive biomarkers of the soil environment [21,80,81,82,83]. In the presented research, DO soil contamination both changed the structure of bacteria and increased their numbers. These elements determined DO’s increasing of the activities of dehydrogenases, catalase, urease, acid phosphatase, alkaline phosphatase, β-glucosidase and arylsulfatase. The response of enzymes from the classes of oxidoreductases and hydrolases to DO hydrocarbons was similar. The same sensitivity of enzymes from these classes to the varying conditions of the soil environment was indicated by Borowik et al. [22] and Wyszkowska et al. [84]. According to Xiaoyan et al. [85], enzymatic activity is dependent on readily available carbon sources.

The increased activities of soil enzymes in the soil exposed to DO pollution, observed in the present study, are due to the stimulated proliferation of soil microorganisms by this petroleum-based product. Many studies [4,73,86] have proved a positive correlation between the abundance of microorganisms in the soil and enzymatic activity. In the present study, DO served as an energy substrate to the microorganisms, hence we could observe its positive effect on the microbial community and, resultantly, on the enhanced activities of soil enzymes, the majority of which are of microbiological origin [85,87]. The positive correlation between the number of microorganisms and the enzymatic activity of soil is also affected by plants, which indirectly affect the synthesis of enzymes by stimulating microorganisms to grow through root secretions [88,89]. In turn, Burns et al. [90] claimed that the preservation of high enzymatic activity in the polluted soil might be due to the sorption of exoenzymes on mineral and organic colloids.

### 4.3. Degradation of Hydrocarbons

Soils polluted with petroleum-based products have increased concentrations of PHAs [91,92]. When they are not severely polluted, the microorganisms colonizing them can lead to their self-treatment [34,93,94]. According to An et al. [95], bacteria representing *Proteobacteria*, *Firmicutes*, *Actinobacteria*, *Chloroflexi* and *Bacteroidetes* are involved in the degradation of hydrocarbons. This process intensifies when aided by plant root secretions, which activate microorganisms of the rhizosphere [92,96,97]. Plants also secrete enzymes into the rhizosphere that degrade soil hydrocarbons. They can also take up hydrocarbons contained in petroleum products [98] and degrade them in their tissues [91]. This system of decomposition of toxic organic compounds is compared to the mammalian liver and is called the “green liver model” [91,99]. According to this model, petroleum compounds undergo transformation in three stages: enzymatic modification, conjugation with low-molecular plant organic compounds and sequestration in a vacuole or cell wall. In the present study, the degradation of DO hydrocarbons was significantly faster in the soil sown with *Dactylis glomerata* than in the non-sown soil. Plants useful in PAH removal from soil also include *Mariscus alternifolius* and *Fimbristylis ferruginea* [86]; *Festuca arundinacea*, *F. elata* and *F. gigantea* [80]; and *Hylotelephium spectabile* [87]. A significant role of plants in the phytoremediation of petroleum-based pollutants was also confirmed by Jing et al. [100] and Iqbal et al. [37]. The transformation of hydrocarbons was also dependent on their structure and properties, i.e., faster degradation was observed for those with a simpler structure than for the more complex ones. The same conclusion was drawn by Wyszkowska et al. [29] and Fatima et al. [36]. The choice of an appropriate plant represents an effective strategy for the remediation of soil polluted with crude oil [36]. Investigations of this type are extremely important, because the same bioremediation methods employed in various types of soil lead to unpredictable and inconsistent effects during the removal of petroleum-based pollutants [101].

The present research focuses on biological tests, such as soil enzymes and various taxa of microorganisms. They were used as indicators for assessing soil contaminated with diesel oil. It has been shown that both microorganisms and soil enzymes react very quickly to changes in the soil. Microorganisms and, above all, enzymes can be used in the biomonitoring of contaminated soils.

## 5. Conclusions

Soil pollution with DO and soil sowing with *Dactylis glomerata* increased population numbers of bacteria and fungi as well as the abundance of the phylum *Proteobacteria*. At the same time, they reduced the relative abundance of *Actinobacteria*, *Acidobacteria*, *Chloroflexi*, *Gemmatimonadetes* and *Firmicutes*, and decreased the values of the ecophysiological diversity index (EP) of the microorganisms tested. In the non-polluted soils, the core microbiome was represented by *Kaistobacter* and *Rhodoplanes*, whereas in DO-polluted soils it was represented by *Parvibaculum* and *Rhodococcus*. Diesel oil stimulated the proliferation of microorganisms and activities of all soil enzymes tested, while *Dactylis glomerata* stimulated the activities of only three enzymes, i.e., dehydrogenases, catalase and arylsulfatase. Additionally, *Dactylis glomerata* proved to be a plant slightly resistant to DO hydrocarbons. With vegetation time proceeding, its resistance increased, which was due to the degradation of individual hydrocarbons. This degradation process was dependent on the chemical structure and chemical properties of the hydrocarbons, and was the faster in the case of the simplest compounds, i.e., xylenes, ethylbenzene, toluene, naphthalene and anthracene, and the slowest in the case of benzo(a)anthracene, benzo(b)fluoranthene and indeno(123-cd)pyrene. Despite the high sensitivity of *Dactylis glomerata* to soil pollution with DO, its usability for the removal of DO hydrocarbons was significant, mainly due to its effect on the soil microbiome and activities of enzymes representing oxidoreductases. *Dactylis glomerata* increased the degradation of gasoline fractions (C_6_–C_12_) by 17%; mineral oil (C_12_–C_35_), by 9%; benzene by 31%; anthracene, by 12%; chrysene, by 38%; benzo[a]anthracene, by 19%; benzo(a)pyrene, by 17%; benzo[b]fluoranthene, by 15%; and benzo(k)fluoranthene, by 18%. It has been shown that tests of microbiological, biochemical and chemical properties can be used in the evaluation of the effects of soil contamination with diesel oil. The methods used are fast and sensitive in the diagnosis of soil contamination with petroleum products, and a combination of all these tests gives a reliable assessment of the state of soils.

## Figures and Tables

**Table 1:** Effect of diesel oil (DO) and *Dactylis glomerata* (Dg) on soil enzyme activity, kg d.m. soil h^−1^.

Object ^1^	Deh	Cat	Ure	Glu	Pac	Pal	Aryl
	µmol TFF	Mol O_2_	mmol N-NH_4_	mmol PNP			
C	0.666 ^c^	0.098 ^d^	0.364 ^b^	0.277 ^c^	1.201 ^b^	0.160 ^b^	0.102 ^d^
DO	3.137 ^b^	0.290 ^c^	2.134 ^a^	0.334 ^a^	1.298 ^a^	0.346 ^a^	0.270 ^b^
Dg	2.920 ^b^	0.727 ^b^	0.235 ^c^	0.265 ^c^	0.930 ^d^	0.122 ^c^	0.244 ^c^
DgDO	5.987 ^a^	1.286 ^a^	0.387 ^b^	0.316 ^b^	1.034 ^c^	0.154 ^b^	0.659 ^a^

^1^ C—non-polluted and non-sown soil, Dg—non-polluted soil sown with *Dactylis glomerata*, DO—non-sown soil polluted with diesel oil, DgDO—soil polluted with diesel oil and sown with *Dactylis glomerata*. Homogeneous groups denoted with letters (a–d) were calculated separately for each enzyme. Deh—dehydrogenases, Cat—catalase, Ure—urease, Glu—β-glucosidase, Pac—acid phosphatase, Pal—alkaline phosphatase, Aryl—arylsulfatase, TFF—triphenyl fomazan, PNP—p-nitrophenol.

**Table 2:** Degradation of hydrocarbons in soil, non-sown and sown, contaminated with diesel oil (DO), %.

**Object**	**C_6_–C_12_**	**C_12_–C_35_**	**Ben**	**EtB**	**Tol**	**Xyl**	**Nap**
NS	65.92 ^b^	52.26 ^b^	44.50 ^b^	98.01 ^a^	97.86 ^b^	98.61 ^a^	97.22 ^b^
S	82.79 ^a^	60.87 ^a^	75.00 ^a^	99.36 ^a^	99.31 ^a^	99.55 ^a^	98.86 ^a^
**Object**	**Ant**	**Chr**	**BaA**	**BaP**	**BbF**	**BkF**	**IP**
NS	80.52 ^b^	13.91 ^b^	18.75 ^b^	27.78 ^b^	18.18 ^b^	22.00 ^b^	10.00 ^b^
S	92.78 ^a^	52.17 ^a^	37.50 ^a^	44.44 ^a^	33.33 ^a^	40.00 ^a^	12.50 ^a^

^1^ C_6_–C_12_—gasoline fractions; C_12_–C_35_—mineral oil; Ben—benzene; EtB—ethylbenzene; Tol—toluene; X—xylene; Nap—naphthalene; Ant—anthracene; Ch—chrysene; BaA—benzo(a)anthracene; BaP—benzo(a)pyrene; BbF—benzo(b)fluoranthene; BkF—benzo(k)fluoranthene; IP—indeno(1,2,3-cd)pyrene. NS—non sown soil, S—sown soil. Homogeneous groups denoted with letters (a,b) were calculated separately for each of the hydrocarbons.

## Supplementary Materials

The following are available online at https://www.mdpi.com/1424-8220/20/12/3362/s1, Table S1: Significance tests carried out using the analysis of variance (ANOVA), Table S2: Covariance matrices for intergroup effects.
