# Supplementary material for: The Role of Dactylis Glomerata and Diesel Oil in the Formation of Microbiome and Soil Enzyme Activity
Source: Sensors (Basel). 2020 Jun 13;20(12):3362. doi: 10.3390/s20123362 (PMC7349710; doi:10.3390/s20123362)
Supplement: Supplementary file 1 [file sensors-20-03362-s001.pdf]

sensors

MDPI

Supplementary Materials

The role of *Dactylis glomerata* and diesel oil in the formation of microbiome and soil enzyme activity

Agata Borowik, Jadwiga Wyszkowska*, Mirosław Kucharski and Jan Kucharski

Table S1 Significance tests carried out using the analysis of variance (ANOVA)

Parameters		F	P
Numbers of microorganisms	Organotrophic bacteria	271.302	0.000
	Actinobacteria	3253.480	0.000
	Fungi	560.760	0.000
Ecophysiological diversity index (EP)	Organotrophic bacteria	21.690	0.000
	Actinobacteria	10.740	0.004
	Fungi	164.600	0.000
Colony development index (CD)	Organotrophic bacteria	98.690	0.000
	Actinobacteria	64.250	0.000
	Fungi	980.340	0.000
Shannon-Weiner's diversity index	Phylum	125.591	0.000
	Class	39.870	0.000
	Order	4.172	0.047
	Family	21.829	0.000
	Genus	192.767	0.000
Enzymes	Dehydrogenases	550.474	0.000
	Catalase	1822.381	0.000
	Urease	26553.260	0.000
	Acid phosphatase	108.870	0.000
	Alkaline phosphatase	492.455	0.000
	Arylsulfatase	2479.480	0.000
	β-glucosidase	79.710	0.000
The yield of grasses	Cut I	171.611	0.000
	Cut II	5.804	0.074
	Cut III	1.316	0.315

F – F-test; P – level of probability.

sensors MDPI

Table S2. Covariance matrices for intergroup effects

Variable factor	Deh	Cat	Ure	Glu	Pac	Pal	Aryl	Org	Act	Fun	CD_{Org}	CD_{Act}	CD_{Fun}	EP_{Org}	EP_{Act}	EP_{Fun}	Shannon Phylum	Shannon Class	Shannon Other	Shannon Family
Cat	0.917**																			
Ure	0.005	-0.383																		
Glu	0.513	0.151	0.759**																	
Pac	-0.374	-0.680*	0.761**	0.591*																
Pal	-0.025	-0.416	0.993**	0.775**	0.814**															
Aryl	0.972**	0.920**	-0.103	0.493	-0.347	-0.112														
Org	0.916**	0.771**	0.107	0.702*	-0.062	0.117	0.954**													
Act	0.896**	0.713**	0.208	0.760**	0.021	0.216	0.928**	0.989**												
Fun	0.931**	0.822**	0.018	0.618*	-0.162	0.025	0.979**	0.985**	0.977**											
CD Org	-0.378	-0.709**	0.902**	0.501	0.860**	0.921**	-0.448	-0.230	-0.121	-0.304										
CD Act	-0.801**	-0.949**	0.551	-0.031	0.680*	0.566	-0.854**	-0.710**	-0.633*	-0.764**	0.813**									
CD Fun	0.558	0.191	0.826**	0.939**	0.453	0.810**	0.473	0.625*	0.698*	0.560	0.536	-0.001								
EP Org	-0.886**	-0.700*	-0.315	-0.632*	0.167	-0.279	-0.803**	-0.793**	-0.811**	-0.793**	0.043	0.561	-0.739**							
EP Act	-0.546	-0.317	-0.360	-0.771**	-0.379	-0.393	-0.622*	-0.760**	-0.807**	-0.736**	-0.165	0.289	-0.659*	0.450						
EP Fun	-0.969**	-0.970**	0.204	-0.361	0.498	0.232	-0.975**	-0.883**	-0.843**	-0.913**	0.566	0.885**	-0.373	0.761**	0.485					
Shannon Phylum	-0.553	-0.189	-0.774**	-0.971**	-0.524	-0.775**	-0.511	-0.690*	-0.767**	-0.624*	-0.506	0.038	-0.969**	0.682*	0.778**	0.392				
Shannon Class	-0.545	-0.192	-0.750**	-0.954**	-0.515	-0.749**	-0.508	-0.683*	-0.760**	-0.618*	-0.480	0.045	-0.948**	0.647*	0.791**	0.397	0.994**			
Shannon Other	-0.143	0.156	-0.769**	-0.677*	-0.554	-0.744**	-0.068	-0.222	-0.311	-0.151	-0.597*	-0.306	-0.733**	0.256	0.491	0.020	0.765**	0.814**		
Shannon Family	0.658*	0.330	0.660*	0.887**	0.333	0.661*	0.600*	0.731**	0.782**	0.683*	0.387	-0.179	0.912**	-0.835**	-0.639*	-0.481	-0.875**	-0.820**	-0.401	
Shannon Genus	0.609*	0.251	0.734**	0.964**	0.470	0.740**	0.569	0.737**	0.804**	0.678*	0.468	-0.106	0.959**	-0.763**	-0.750**	-0.437	-0.967**	-0.934**	-0.588*	0.966**

**significant values at $p < 0.01$

*significant values at $p < 0.05$
